# Quality of Life After Partial or Whole-Breast Irradiation in Breast-Conserving Therapy for Low-Risk Breast Cancer: 1-Year Results of a Phase 2 Randomized Controlled Trial

**DOI:** 10.3389/fonc.2021.738318

**Published:** 2021-09-15

**Authors:** Yu-Chun Song, Guang-Yi Sun, Hui Fang, Yu Tang, Yong-Wen Song, Chen Hu, Shu-Nan Qi, Bo Chen, Hao Jing, Yuan Tang, Jing Jin, Yue-Ping Liu, Ning-ning Lu, Ye-Xiong Li, Shu-Lian Wang

**Affiliations:** ^1^Department of Radiation Oncology, National Cancer Center/National Clinical Research Center for Cancer/Cancer Hospital, Chinese Academy of Medical Sciences and Peking Union Medical College, Beijing, China; ^2^Clinical Trials Center, National Cancer Center/National Clinical Research Center for Cancer/Cancer Hospital, Chinese Academy of Medical Sciences and Peking Union Medical College, Beijing, China; ^3^Division of Biostatistics and Bioinformatics, Sidney Kimmel Comprehensive Cancer Center, Johns Hopkins University School of Medicine, Baltimore MD, United States

**Keywords:** breast neoplasm, breast-conserving surgery, partial breast irradiation, whole-breast irradiation, quality of life

## Abstract

**Purpose:**

To report patients’ quality of life (QoL) at 1 year in a phase 2 randomized trial comparing partial breast irradiation (PBI) with whole-breast irradiation (WBI) after breast-conserving surgery (BCS) for breast cancer.

**Methods:**

Women aged ≥ 45 years with low-risk breast cancer after BCS were randomly assigned (1:1) to receive PBI (40 Gy in 10 fractions over 2 weeks) or WBI (43.5 Gy in 15 fractions over 3 weeks). The primary endpoint—the incidence of toxicities of grade 2 or higher—will be reported when participants complete 5 years of follow-up. QoL was assessed at baseline (T0), at the end of radiotherapy (RT) (T1), 6 months (T2) and 1 year (T3) after RT by using the EORTC QLQ-C30 and QLQ-BR23 questionnaires. We calculated the scores for all QOL subscales and differences in mean scores were compared. This study was registered at ClinicalTrials.gov (NCT03583619).

**Results:**

Between June 2017 and January 2019, 140 women were randomly assigned to receive PBI or WBI (n = 70 per group). Fifty-nine and 56 patients treated with PBI and WBI, respectively, were eligible for the QoL analysis. There were no significant differences in any subscale scores at T0, T1, T2, or T3 between the PBI and WBI arms. The scores for most QoL subscales that were influenced by RT recovered to a similar or better level relative to T0 scores within 1 year after RT, except for the scores of the dyspnea subscale. Longitudinal analysis showed that time since RT had a significant impact on physical functioning, role functioning, social functioning, fatigue, pain, dyspnea, financial difficulties, body image, and breast and arm symptoms.

**Conclusion:**

PBI using the intensity-modulated RT affords QoL comparable to that provided by WBI. Most QoL subscale scores that were influenced by RT recovered to a similar or better level relative to baseline scores within 1 year after RT.

## Introduction

According to the latest data reported by International Agency for Research on Cancer in 2020, breast cancer is the most common cancer worldwide (1). Whole-breast irradiation (WBI) with or without a tumor bed boost is the standard treatment for patients after breast-conserving surgery, which offers excellent local control and overall survival equivalent to that afforded by modified radical mastectomy (2, 3). Approximately 80% of sites of local recurrence are around the tumor bed, and the relapse rate for other quadrants is similar to the tumor incidence in the contralateral breast (4). Therefore, partial breast irradiation (PBI), wherein only the tumor bed and the surrounding region are irradiated with a hypofractionation regimen, is an alternative approach to WBI for the management of low-risk early-stage breast cancer. Compared to WBI, PBI has the advantages of a shorter treatment period, lower cost, and exposure of normal tissues of a lower dose.

Several large, randomized studies have shown that PBI provides long-term locoregional control and survival comparable to those afforded by WBI (5–9). However, the appropriate radiation technique and dose fractionation for PBI are not well defined. In the Florence trial, 30 Gy in five daily fractions was used for PBI with intensity-modulated radiotherapy (IMRT), and treatment-related toxicity and cosmesis outcomes were found to be significantly in favor of PBI (8). On the other hand, in the RAPID trial, 38.5 Gy in 10 fractions and 3.85 Gy bid was used for PBI with 3D conformal radiotherapy (3DCRT) or IMRT. Furthermore, it was found that this approach was associated with a higher incidence of late toxicity and worse cosmesis outcomes in the PBI arm than in the WBI arm (9). In addition, more attention should be paid to improving the quality of life (QoL) for women with early-stage breast cancer, because of their excellent long-term survival. Some studies have compared the QoL between WBI and PBI using different techniques (10–13); however, only one randomized study involved the use of external-beam radiation (11) and only few such retrospective studies have been conducted in China (14). We initiated a randomized phase 2 trial to primarily compare the toxicities between PBI and WBI in Chinese women. In the PBI arm, we explored a new regimen of 40 Gy in 10 daily fractions delivered with tangential IMRT. The purpose of the present analysis is to evaluate the QoL at 1 year.

## Materials and Methods

### Study Design and Participants

This was a randomized, controlled, phase 2 trial for patients with low-risk early-stage breast cancer performed at our hospital between 2017 and 2019. The inclusion criteria were set up as follows: age between 45 and 75 years; life expectancy higher than 5 years; presence of histologically confirmed invasive ductal carcinoma (grade 1-2), mucinous carcinoma, papillary carcinoma, or tubular carcinoma with the maximum tumor diameter being ≤3.0 cm; or histologically confirmed ductal carcinoma *in situ* (low-medium grade) with the maximum tumor diameter being ≤2.5 cm; pN0 (for patients with invasive carcinoma, either an axillary dissection with minimum of six nodes in the specimen or a negative sentinel node was required); presence of a unifocal tumor (confirmed by MRI); negative lymphovascular invasion; positive estrogen receptor (ER) or progesterone receptor (PR) status; negative resection margins of ≥2 mm; surgical clips placed in the tumor bed; and enrollment date less than 12 weeks after breast-conserving surgery or less than 8 weeks after adjuvant chemotherapy. The exclusion criteria were as follows: presence of disease classified as stage II-IV per the 7th edition of the American Joint Committee on Cancer (AJCC); invasive micropapillary carcinoma, lobular carcinoma in situ, invasive lobular carcinoma, or Paget’s disease alone; previous oncoplastic surgery of the affected breast; neoadjuvant chemotherapy or hormonal therapy; presence of simultaneous contralateral breast cancer; previous ipsilateral breast or thorax irradiation; or active collagen vascular disease. All patients provided written informed consent. The study protocol was approved by the local ethics committee (Approval Number 17-139/1395) and registered at ClinicalTrials.gov (NCT 03583619).

### Randomization and Masking

The patients enrolled were randomly assigned (1:1) to receive PBI or WBI without stratification by simple randomization according to a prescribed computer-generated central randomization schedule. Patients and investigators were not masked to treatment allocation.

### Procedures

All patients had undergone breast-conserving surgery, and clips were placed at the borders of the tumor bed during the operation. Adjuvant systemic therapy was performed before or after radiotherapy (RT) if indicated according to the National Comprehensive Cancer Network guideline. Hormonal therapy was recommended to patients with positive ER and/or PR tumors. Patients with a positive human epidermal growth factor receptor 2 (HER2) status were recommended to receive anti-HER2-targeted therapy.

All patients underwent computed tomography (CT) simulation with a slice thickness of 5 mm. Patients in the WBI arm received a total dose of 43.5 Gy in 15 daily fractions over 3 weeks. The clinical target volume (CTV) included the whole breast and the fascia of the pectoralis major, limited to 5 mm from the skin surface. Planning target volume (PTV) was determined by extending a 6-mm margin in all directions to the CTV and limited to 5 mm from the skin surface.

The patients in the PBI arm received a total dose of 40 Gy in 10 daily fractions over 2 weeks. The tumor bed was contoured according to the surgical clips, seroma, and postoperative changes, and CTV was expanded from the tumor bed with a 1.5-cm margin three-dimensionally and limited to 5 mm from the skin surface. PTV was obtained by adding a 6-mm margin in all directions to the CTV and was limited to 5 mm from the skin surface.

The ipsilateral breast, contralateral breast, lung, heart, left anterior descending artery, and cord were contoured, and the ipsilateral breast outside the PTV (Breast ‐ PTV) was constructed for dose constraints. WBI and PBI were both delivered using tangential field-in-field IMRT, with the prescribed dose covering 95 percent of the PTV. To achieve better homogeneity and conformality, the treatment plans were required to meet the following constraints: in the PBI arm, PTV: V43Gy < 5%, Dmax < 44 Gy; Breast ‐ PTV: V20Gy < 60%, V40Gy < 35%, Dmax < 44 Gy; ipsilateral lung: mean dose < 5 Gy, V10Gy < 20%; contralateral lung: V5Gy < 10%; heart: mean dose < 2.5 Gy; contralateral breast: V4Gy < 5%. In the WBI arm, PTV: V47Gy < 5%; ipsilateral lung: mean dose < 10 Gy, V20Gy < 20%; contralateral lung: V5Gy < 10%; heart: mean dose < 5 Gy; contralateral breast: V4Gy < 5%. The treatment plan was developed using the Pinnacle treatment planning system.

### Quality of Life Assessment

QoL was evaluated using the EORTC QLQ-C30 version 3.0 (15), and breast cancer-specific module QLQ-BR23 questionnaires (16). The questionnaires have good reliability, validity, and responsiveness in patients with cancer, and the Chinese version has been well validated (17–20). All participants were asked to complete these two questionnaires before RT (baseline, T0), at the end of RT (time 1, T1), 6 months after RT (time 2, T2), and 1 year after RT (time 3, T3).

The EORTC QLQ-C30 consists of 30 questions, including those on the global health status (GHS) scale, five functional scales (physical, role, emotional, cognitive, and social functioning), three multi-term symptom subscales (fatigue, pain, nausea and vomiting), and six single-term symptom subscales (dyspnea, insomnia, appetite loss, constipation, diarrhea, and financial difficulties). The EORTC QLQ-BR23 has 23 questions, including those on four functional subscales (body image, sexual functioning, sexual enjoyment, and future perspective) and four symptom subscales (systemic therapy side effects, breast symptoms, arm symptoms, and upset by hair loss). All questions had the same response categories and responses were scored on a scale of 1-4 (not at all, a little, quite a bit, and very much), except for the GHS subscale, the responses for which were scored on a scale of 1-7 (very bad to excellent). The responses for all QoL subscales were scored according to the EORTC scoring manual and converted to standard scores ranging from 0 to 100. For the functional and GHS subscales, a higher score indicates a higher level of functioning or health status. For the symptom subscales, a higher score indicates a higher level of symptoms or problems and worse QoL. We hypothesized that the QoL of the PBI arm was noninferior to that of the WBI arm.

### Statistical Analysis

Chi-squared or Mann-Whitney U test was used to compare characteristics between the PBI and WBI arms. The scores for all QOL subscales were calculated, and the difference in the mean scale scores were compared, as frequently as that reported previously in the literature. The Mann-Whitney U test was used to compare the difference between the two arms owing to the non-normal distribution of data. The Wilcoxon-signed rank test was used to examine the difference among T1, T2, T3, and baseline T0, respectively, in each arm. A longitudinal analysis of QoL changes over time and between arms was performed with generalized estimating equations (GEE) with log link function for mean scores because of the non-normal distribution of data. Given that the missing questionnaires were random and not influenced by other factors, the missing data were considered missing completely at random (MCAR). After several tries, autocorrelation (AR) was selected as the working correlation matrix, which had the minimum value of quasi‐likelihood under the independence model criterion (QIC). Further, an interaction term between time and treatment was used to assess if the changes in the mean scale scores over time were statistically different between the two arms. Longitudinal analyses were mainly focused on selected subscales of QLQ-C30 (GHS, physical functioning, role functioning, emotional functioning, social functioning, fatigue, pain, dyspnea, and financial difficulties), and QLQ-BR23 (body image, future perspective, breast symptoms, and arm symptoms). All analyses were performed according to the treatment received (per-protocol population).

A two-sided P-value of <0.01 was considered significant because multiple testing could lead to type I error (11, 21–23). Clinical significance was set according to the Osoda method: a difference of at least 10 points was considered a minimal clinically meaningful change, of 10-20 points was considered a moderate difference, and of more than 20 points was considered a large difference (24). IBM-SPSS version 22 was used for statistical analysis.

## Results

**Figure 1:**
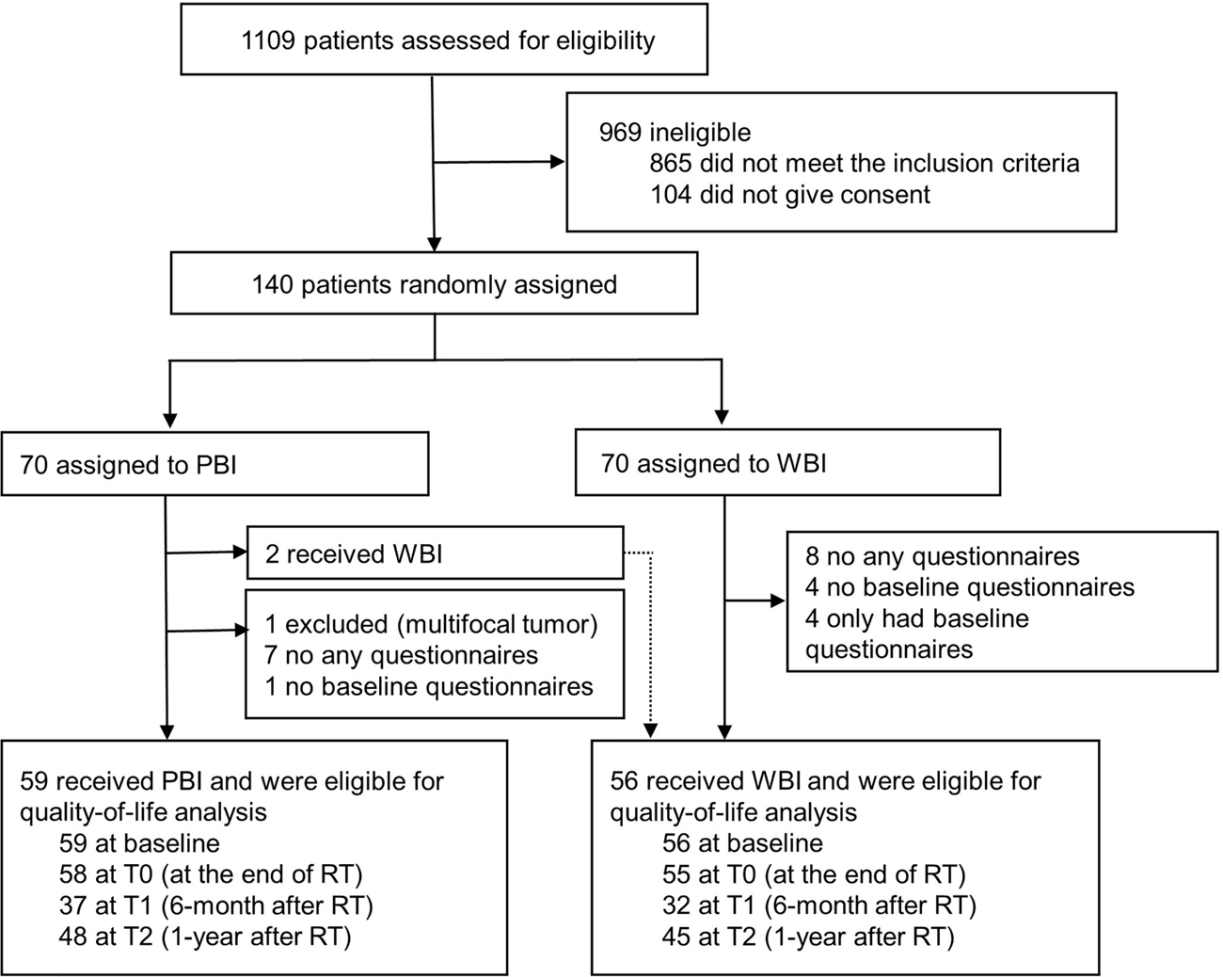
CONSORT diagram. PBI, partial breast irradiation; WBI, whole-breast irradiation.

From June 2017 to January 2019, 140 patients with early-stage breast cancer were randomly assigned to the PBI (n = 70) or WBI arm (n = 70). After one patient was excluded due to a multifocal tumor, 67 and 70 women in the PBI and WBI arms, respectively, received the allocated treatment, while 2 patients in the PBI arm underwent WBI. Finally, 115 participants who completed the QoL questionnaires at baseline and at least one other timepoint were eligible for the present analysis, including 59 (86.8%) patients who received PBI and 56 (77.8%) who received WBI (**Figure 1**).

The clinical and treatment-related characteristics were well balanced between the two arms (**Table 1**). All patients underwent lumpectomy and sentinel lymph node biopsy, except for 2 patients who were treated with lumpectomy and axillary lymph node dissection. In all, 23 (20%) patients received chemotherapy with a median of 4 cycles (range, 3-10), mostly with taxane or anthracycline-based regimens. All participants had a positive ER and/or PR status and received hormonal therapy. Of the 8 patients with positive HER2 disease, seven received trastuzumab. Three patients in the WBI arm received a simultaneous integrated tumor bed boost at a total of 49.5 Gy in 15 fractions at the discretion of the attending physicians. The median overall RT period was 14 (range, 11-17) days for the PBI arm and 21 (range, 18-23) days for the WBI arm.

**Table 1:** Clinical characteristics and treatments between the PBI and WBI arms.

	PBI arm (n = 59, %)	WBI arm (n = 56, %)	P
Age (years)			
Median (range)	54 (45-69)	53.5 (46-71)	0.644
Menopausal status			
Premenopausal	19 (32.2)	13 (23.2)	0.550
Perimenopausal	5 (8.5	6 (10.7)	
Postmenopausal	35 (59.3)	37 (66.1)	
T stage			
pTis	2 (3.4)	3 (5.3)	0.330
pT1	51 (86.4)	51 (91.1)	
pT2	6 (10.2)	2 (3.6)	
HER2 status			
Positive	4 (6.8)	4 (7.1)	0.231
Negative	55 (93.2)	50 (88.3)	
Unknown	0	2 (3.6)	
Chemotherapy			
No	47 (79.7)	43 (76.8)	0.709
Yes	12 (19.7)	13 (23.2)	
Trastuzumab therapy			
No	56 (94.9)	52 (92.9)	0.943
Yes	3 (5.1)	4 (7.1)	
Surgery–radiotherapy interval (weeks)			
Median (range)	8.4 (4.1-29.4)	7.9 (4.3-29.7)	0.288

HER2, human epidermal growth factor receptor 2; PBI, partial breast irradiation; WBI, whole-breast irradiation.

**Figure 2:**
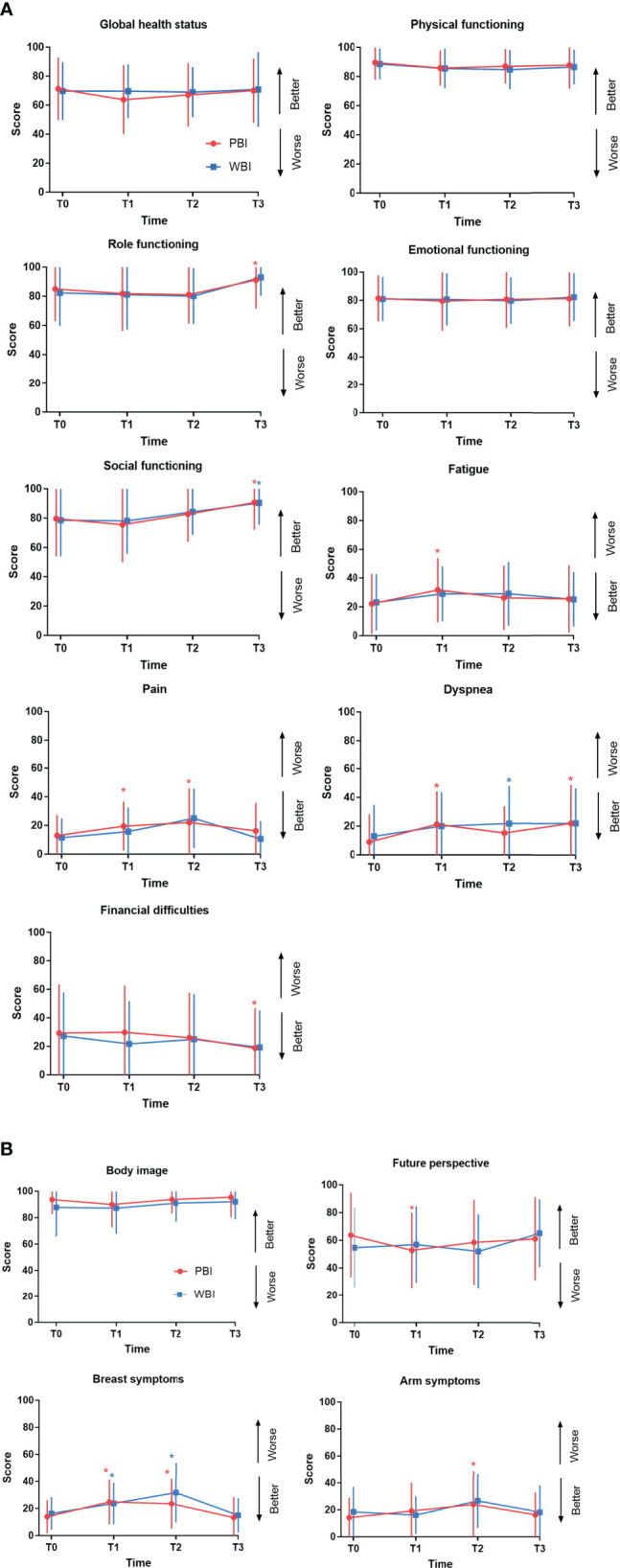
Quality of life changes over time in the PBI and WBI arm determined using the QLQ-C30 **(A)** and QLQ-BR23 **(B)** questionnaires. These graphs show changes in the mean QoL scores over time for each domain. Error bars represent standard deviation (SD). *Time points at which differences from baseline were significant at P < 0.01 level. PBI, partial breast irradiation; WBI, whole-breast irradiation; QoL, quality of life.

The comparison of mean scores and standard deviation (SD) of EORTC QLQ-C30 and QLQ-BR23 between the two arms at different timepoints is shown in **Tables 2** and **3**. No significant differences at T0 or other time points were found between the PBI and WBI arms. Longitudinal changes in the mean scores of the selected subscales and the differences between baseline T0 and other time points in each arm for QLQ-C30 are presented in **Figure 2A**. The role functioning scores significantly improved at T3 compared to the scores at T0 in the PBI arm; the scores also improved in the WBI arm but not significantly. The social functioning scores was significantly better at T3 than at T0 in both arms. The scores for fatigue increased from T0 to T1 in both arms, and the difference in the PBI arm was significant; nevertheless, the scores recovered at T2. The pain scores were significantly higher at T1 and T2 than at T0 but recovered at T3 in the PBI arm. The scores for financial difficulties significantly decreased at T3 compared to those at T0 in the PBI arm. The dyspnea scores were significantly higher at T1 and T3 than at T0 in the PBI arm. On the other hand, the scores were significantly higher at T2 than at T0 in the WBI arm. The scores for GHS, physical functioning, and emotional functioning were generally stable during the follow-up period in both arms. The longitudinal changes in the mean scores of the selected subscales for QLQ-BR23 are shown in **Figure 2B**. A significant deterioration of future perspective was recorded at T1 compared to that at T0; however, the scores recovered at T2 and T3 in the PBI arm. Breast symptom scores significantly increased at T1 and T2 in both the arms but decreased at T3, which was comparable to baseline scores. The arm symptom scores significantly increased at T2 in the PBI arm and recovered to a level similar to the baseline level at T3.

**Table 2:** Mean scores (and SD) of EORTC QLQ-C30 at T0, T1, T2, and T3 between the two arms.

	PBI arm	WBI arm	Difference in means	
	Mean (SD)	Mean (SD)	Estimate (95% CI)	P
Global health status				
T0	71.3 (21.5)	69.8 (19.9)	1.5 (-6.1;9.2)	0.744
T1	63.9 (23.9)	69.7 (18.5)	-5.8 (-13.7;2.2)	0.282
T2	67.1 (21.9)	69.0 (17.4)	-1.9 (-11.5;7.7)	0.816
T3	70.1 (22.2)	70.9 (25.8)	-0.8 (-10.7;9.1)	0.695
Physical functioning				
T0	89.6 (11.8)	88.6 (10.5)	1.0 (-3.1;5.1)	0.406
T1	85.9 (12.1)	85.5 (13.5)	0.4 (-4.4;5.2)	0.986
T2	87.0 (11.9)	84.8 (13.4)	2.2 (-3.8;8.3)	0.544
T3	87.8 (16.1)	86.5 (11.9)	1.3 (-4.6;7.1)	0.251
Role functioning				
T0	85.0 (22.0)	82.4 (22.8)	2.6 (-5.7;10.9)	0.466
T1	81.9 (25.6)	81.2 (23.8)	0.7 (-8.5;9.9)	0.646
T2	81.1 (19.7)	80.2 (19.1)	0.9 (-8.5;10.2)	0.778
T3	91.3 (19.7)	93.0 (12.6)	-1.6 (-8.5;5.2)	0.729
Emotional functioning				
T0	81.5 (16.3)	81.0 (15.5)	0.5 (-5.3;6.4)	0.780
T1	79.6 (20.9)	80.8 (18.4)	-1.2 (-8.5;6.2)	0.916
T2	80.9 (20.3)	79.9 (16.5)	0.9 (-8.1;9.9)	0.606
T3	81.3 (19.8)	82.4 (17.0)	-1.2 (-8.9;6.5)	0.975
Cognitive functioning				
T0	88.1 (18.6)	86.6 (13.6)	1.5 (-4.5;7.6)	0.193
T1	81.3 (20.0)	87.3 (12.8)	-6.0 (-12.3;0.35)	0.167
T2	79.7 (18.9)	83.3 (16.9)	-3.6 (-12.3;5.1)	0.444
T3	78.1 (19.8)	82.6 (17.0)	-4.5 (-12.1;3.2)	0.274
Social functioning				
T0	79.7 (25.7)	78.6 (24.6)	1.1 (-8.2;10.4)	0.549
T1	75.6 (25.6)	78.2 (22.4)	-2.6 (-11.6;6.4)	0.892
T2	82.9 (19.0)	84.4 (15.8)	-1.5 (-10.0;7.0)	0.866
T3	90.6 (18.5)	90.4 (14.9)	0.3 (-6.7;7.2)	0.595
Fatigue				
T0	22.2 (20.9)	23.2 (19.5)	-1.0 (-8.5;6.5)	0.709
T1	31.8 (22.5)	29.1 (19.1)	2.7 (-5.1;10.5)	0.544
T2	26.4 (22.4)	29.2 (22.2)	-2.7 (-13.5;8.0)	0.610
T3	25.5 (23.4)	25.2 (18.9)	0.3 (-8.5;9.1)	0.727
Nausea–vomiting				
T0	3.4 (7.4)	1.8 (6.1)	1.6 (-0.9;4.1)	0.142
T1	4.9 (9.4)	5.5 (12.4)	-0.6 (-4.7;3.5)	0.842
T2	1.8 (5.2)	4.2 (9.5)	-2.4 (-6.0;1.2)	0.315
T3	3.5 (8.4)	2.6 (7.9)	0.9 (-2.5;4.2)	0.467
Pain				
T0	13.0 (14.2)	11.6 (13.5)	1.4 (-3.7;6.5)	0.611
T1	19.5 (17.1)	15.8 (16.8)	3.8 (-2.5;10.1)	0.210
T2	22.1 (23.9)	25.0 (20.7)	-2.9 (-13.8;7.9)	0.323
T3	16.3 (19.6)	10.7 (12.4)	5.6 (-1.2;12.4)	0.262
Dyspnea				
T0	9.0 (19.4)	13.0 (21.7)	-4.1 (-11.7;3.5)	0.224
T1	21.3 (23.1)	20.0 (23.7)	1.3 (-7.5;10.0)	0.710
T2	15.3 (18.6)	21.9 (26.2)	-6.6 (-17.4;4.3)	0.381
T3	22.2 (26.9)	22.2 (24.6)	0 (-10.6;10.6)	0.852
Insomnia				
T0	30.5 (32.3)	30.4 (32.0)	0.2 (-11.7;12.0)	0.998
T1	32.2 (31.8)	35.8 (31.3)	-3.6 (-15.4;8.2)	0.488
T2	37.8 (31.6)	46.9 (39.6)	-9.0 (-26.1;8.1)	0.378
T3	29.2 (32.0)	33.3 (28.4)	-4.2 (-16.7;8.3)	0.312
Appetite loss				
T0	12.4 (22.2)	4.8 (13.4)	7.7 (0.8;14.5)	0.029
T1	13.8 (25.8)	13.3 (21.8)	0.5 (-8.5;9.4)	0.790
T2	9.9 (19.0)	11.5 (21.8)	-1.5 (-11.3;8.3)	0.879
T3	6.3 (14.8)	5.9 (14.7)	0.3 (-5.8;6.4)	0.890
Constipation				
T0	11.3 (21.1)	11.3 (20.4)	0 (-7.6;7.6)	0.906
T1	8.0 (20.0)	8.5 (15.9)	-0.4 (-7.2;6.3)	0.475
T2	9.9 (17.3)	9.4 (15.2)	0.5 (-7.4;8.4)	0.975
T3	15.3 (22.8)	10.4 (22.3)	4.9 (-4.4;14.2)	0.143
Diarrhea				
T0	5.6 (15.4)	1.2 (6.2)	4.5 (-0.1;8.8)	0.056
T1	7.5 (18.8)	6.1 (14.5)	1.4 (-4.9;7.8)	0.870
T2	4.5 (14.0)	2.2 (8.2)	2.4 (-3.2;8.0)	0.490
T3	6.9 (19.4)	5.2 (14.1)	1.8 (-5.3;8.8)	0.833
Financial difficulties				
T0	29.4 (34.3)	27.4 (30.6)	2.0 (-10.0;14.0)	0.950
T1	29.9 (32.9)	21.8 (30.2)	8.1 (-3.7;19.9)	0.154
T2	26.1 (31.6)	25.0 (31.7)	1.1 (-14.1;16.3)	0.834
T3	18.8 (28.3)	19.3 (26.1)	-0.5 (-11.7;10.7)	0.782

T0, before radiotherapy; T1, at the end of radiotherapy; T2, at 6 months after radiotherapy; T3, at 1 year after radiotherapy; PBI, partial breast irradiation; WBI, whole-breast irradiation.

**Table 3:** Mean scores (and SD) of EORTC QLQ-BR23 at T0, T1, T2, and T3 between the two arms.

	PBI arm	WBI arm	Difference in means	
	Mean (SD)	Mean (SD)	Estimate (95% CI)	P value
Body image				
T0	93.8 (10.9)	87.9 (22.0)	5.8 (-0.5;12.2)	0.162
T1	90.1 (17.3)	87.3 (19.5)	2.8 (-4.1;9.7)	0.207
T2	93.9 (10.7)	91.1 (14.0)	2.8 (-3.2;8.7)	0.523
T3	95.5 (15.2)	92.2 (13.3)	3.3 (-2.6;9.1)	0.087
Sexual functioning				
T0	15.5 (18.8)	13.7 (21.6)	1.8 (-5.6;9.3)	0.344
T1	12.6 (17.5)	10.9 (15.7)	1.7 (-4.5;8.0)	0.655
T2	19.8 (23.5)	10.9 (14.4)	8.9 (-0.7;18.4)	0.150
T3	16.3 (22.9)	11.9 (16.9)	4.5 (-3.9;12.8)	0.540
Sexual enjoyment				
T0	21.9 (22.8)	17.3 (23.8)	4.6 (-7.6;16.7)	0.369
T1	22.2 (26.5)	16.7 (21.6)	5.6 (-8.6;19.7)	0.525
T2	33.3 (29.1)	33.3 (23.6)	0 (-19.4;19.4)	0.906
T3	26.9 (28.3)	21.3 (27.0)	5.6 (-10.0;21.2)	0.465
Future perspective				
T0	63.8 (30.5)	54.8 (28.7)	9.1 (-1.9;20.0)	0.091
T1	52.9 (27.2)	57.0 (27.7)	-4.1 (-14.3;6.2)	0.524
T2	58.6 (30.8)	52.1 (26.7)	6.5 (-7.5;20.4)	0.251
T3	61.1 (30.2)	65.2 (24.6)	-4.1 (-15.5;7.3)	0.679
Systemic therapy side effects				
T0	16.9 (12.6)	14.6 (11.4)	2.3 (-2.1;6.8)	0.326
T1	20.9 (15.7)	18.4 (12.7)	2.4 (-2.9;7.7)	0.575
T2	19.2 (14.7)	20.2 (13.8)	-1.1 (-8.0;5.8)	0.744
T3	17.7 (14.0)	19.3 (13.0)	-1.6 (-7.2;4.0)	0.650
Breast symptoms				
T0	14.0 (12.2)	16.4 (12.1)	-2.4 (-6.9;2.1)	0.188
T1	24.9 (16.5)	23.8 (15.3)	1.1 (-4.9;7.0)	0.775
T2	23.6 (18.4)	31.8 (22.0)	-8.1 (-17.8;1.6)	0.116
T3	13.4 (15.1)	15.0 (12.6)	-1.6 (-7.4;4.1)	0.307
Arm symptoms				
T0	14.3 (14.9)	18.5 (18.5)	-4.1 (-10.3;2.0)	0.277
T1	19.2 (21.1)	16.2 (14.0)	3.0 (-3.7;9.7)	0.912
T2	24.0 (24.9)	26.7 (20.1)	-2.7 (-13.7;8.3)	0.337
T3	16.4 (16.7)	18.3 (20.1)	-1.8 (-9.4;5.8)	0.905
Upset by hair loss				
T0	45.6 (37.2)	38.6 (40.5)	7.0 (-18.6;32.6)	0.707
T1	30.0 (30.4)	33.3 (33.3)	-3.3 (-24.0;17.3)	0.813
T2	33.3 (29.8)	33.3 (21.1)	0 (-21.5;21.5)	0.865
T3	23.2 (27.4)	21.3 (28.7)	1.9 (-14.5;18.2)	0.909

T0, before radiotherapy; T1, at the end of radiotherapy; T2, at 6 months after radiotherapy; T3, at 1 year after radiotherapy; PBI, partial breast irradiation; WBI, whole-breast irradiation.

The results of the longitudinal analysis are presented in **Table 4**. Treatment and its interaction with time (treatment × time) had no significant impact on the selected subscales, suggesting there was no significant difference in the changes of selected subscale scores over time between the two arms. Time since RT had a significant impact on physical functioning, role functioning, social functioning, fatigue, pain, dyspnea, financial difficulties, body image, breast symptoms, and arm symptoms when the scale scores were compared at different timepoints. For example, the scores for role functioning at T3 were significantly higher than those at T0 for all patients in both arms, and there was a significant difference among the scores at all four timepoints. There was no post-surgery relapse or death after a median follow-up time of 25.9 months (range, 18.8-41.8 months).

**Table 4:** Summary of generalized estimating equation for quality-of-life outcomes, P values.

Domain	Treatment	Time				Treatment × time
		T1 *vs.* T0	T2 *vs.* T0	T3 *vs.*T0	T3 *vs.* T2 *vs.* T1 *vs.* T0	
Global health status	0.254	0.083	0.153	0.842	0.609	0.403
Physical functioning	0.582	0.005(0.961)	0.015	0.287	0.034	0.986
Role functioning	0.891	0.307	0.346	0.001 (1.099)	<0.001	0.750
Emotional functioning	0.890	0.488	0.949	0.918	0.860	0.865
Social functioning	0.944	0.259	0.064	<0.001 (1.146)	<0.001	0.708
Fatigue	0.944	<0.001 (1.342)	0.029	0.191	<0.001	0.554
Pain	0.178	<0.001 (1.448)	<0.001 (1.840)	0.479	<0.001	0.348
Dyspnea	0.565	<0.001 (1.864)	0.001 (1.790)	<0.001 (2.052)	<0.001	0.437
Financial difficulties	0.736	0.328	0.576	<0.001 (0.678)	0.002	0.319
Body image	0.111	0.152	0.872	0.029	0.001	0.615
Future perspective	0.722	0.054	0.307	0.146	0.013	0.011
Breast symptoms	0.385	<0.001 (1.619)	<0.001 (1.811)	0.465	<0.001	0.053
Arm symptoms	0.511	0.325	<0.001 (1.576)	0.590	<0.001	0.058

Values in parentheses are the mean ratios of QoL scale scores at different timepoints when P value < 0.01.

T0, before radiotherapy; T1, at the end of radiotherapy; T2, at 6 months after radiotherapy; T3, at 1 year after radiotherapy.

## Discussion

The present analysis provides valuable and reliable information about the impact of PBI and WBI on patients’ quality of life by collecting longitudinal data during a 1-year period. We found that PBI using IMRT provided QoL comparable to that afforded by WBI at each timepoint. At the 1-year follow-up after RT, most QoL subscale scores influenced by RT recovered to a similar or better level compared to the baseline level.

Most studies have reported better or similar QoL for patients treated with PBI compared to those treated with WBI, although different techniques have been used for PBI including intraoperative radiation (IORT), interstitial brachytherapy, and external-beam RT (11, 12, 22), shown in **Table 5**. In the GEC-ESTRO trial, it was reported that PBI using multicatheter brachytherapy did not result in worse QoL compared to that achieved with WBI, and the scores for breast symptoms was significantly better in the PBI arm (12). Corica et al. analyzed the effect of single-dose IORT *versus* WBI on QoL in the TARGIT-A trial. They found that the patients treated with IORT had better breast-related QoL and fewer breast symptoms than did those treated with WBI during the 5-year follow-up (22). Meattini et al. reported the QoL in the phase 3 Florence trial, which indicated that at the end of RT and 2 years after RT, women who underwent PBI (30 Gy in five fractions over 2 weeks using IMRT) experienced significantly better QoL than did those treated with WBI, as shown by the scores of most subscales such as GHS; physical, role, and emotional functioning; body image; future perspective; and breast and arm symptoms (11). In contrast, the RTOG 0413 trial, which investigated the efficacy and safety of PBI delivered with brachytherapy or 3DCRT compared with WBI, reported the QoL results at the American Society of Clinical Oncology annual meeting in 2019 (25), shown in **Table 5**. Patients with and without chemotherapy were analyzed separately. In the no-chemotherapy group, PBI-treated patients experienced significantly less fatigue at 3 years after RT, whereas in the chemotherapy group, the PBI arm had significantly more fatigue than the WBI arm. A retrospective study in China (**Table 5**), which compared the QoL afforded by PBI with that afforded by WBI using the FACT-B questionnaire, showed that the PBI-treated patients (using 3DCRT with a total dose of 34 Gy in 10 fractions over 1 week, twice per day) had similar QoL in terms of the physical, functional, and social domains, and breast-specific concerns compared to WBI-treated patients. However, the PBI arm had a worse emotional response than the WBI arm (14). In our study, no clinically and statistically significant difference was found in the various QoL subscale scores between the PBI and WBI arms. The comparable QoL results might be related to the better dose conformality and homogeneity with IMRT, the benefits of a once-daily regimen, and the smaller breast size in Chinese women.

**Table 5:** Summary of QoL comparisons between APBI and WBI.

Study	n	Technique for APBI	Treatment	QoL results (APBI compared with WBI)
GEC-ESTRO (12)	1184	IBT	WBI (50 Gy in 25 fr + 10-Gy boost)	Better QoL regarding breast symptoms and arm symptoms
			APBI (32 Gy in 8 fr or 30.1 Gy in 7 fr [HDR] or 50 Gy in 0.6–0.8 Gy per pulse [PDR])	
TARGIT-A (22)	3451	IORT	WBI (50 Gy in 25 fr +/– 10-Gy boost),	Better QoL regarding breast symptoms
			APBI (20 Gy to the 90% isodose)	
Florence IMRT (11)	520	IMRT	WBI (50 Gy in 25 fr + 10-Gy boost)	Better QoL regarding most subscales, such as GHS, physical, role, and emotional functioning at al
			PBI (30 Gy in 5 fr, 2wks)	
RTOG 0413 (25)	4216	IBT, 3DCRT	WBI (50 Gy in 25 fr +/– 10-Gy boost)	Less fatigue in the no-chemotherapy group, and more fatigue in the chemotherapy group.
			APBI (34–38.5 Gy in 10 fr, bid)	
A retrospective study in China (14)	84	3DCRT	WBI (48.6-50Gy in 25-27fr + 10-Gy boost)	Worse QoL regarding emotional response
			APBI (34 Gy in 10 fr, bid)	

Present study	115	IMRT	WBI (43.5 Gy in 15 fr +/– 8.7-Gy boost)	Similar QoL
			PBI (40 Gy in 10 fr, 2wks)	

APBI, accelerated partial breast irradiation; QoL, quality of life; IBT, interstitial brachytherapy; WBI, whole breast irradiation; PBI, partial breast irradiation; fr, fractions; EBRT, external beam–based radiotherapy; IORT, intraoperative radiation therapy; wks, weeks; GHS, global health status.

In our study, patients assigned to the PBI arm received a prescribed dose of 40 Gy in 10 fractions once per day over 2 weeks *via* IMRT, which is different from the RT regimens in other clinical trials. The regimen of 38.5 Gy in 10 fractions twice per day was most used for external-beam PBI, such as in the RAPID and RTOG 0413 trials. However, the RAPID trial showed that PBI resulted in more late toxicities than did WBI (9). External-beam PBI twice per day might not be an appropriate schedule for the modality. Some studies suggested that an interval between external beam fractions of 6 h was not enough to repair the sublethal damage to normal tissues (26), whereas late radiation effects would be fewer when the interval was 24 hours or more (27–29). In addition, delivering PBI twice per day did not save medical resources, and it might increase fatigue in patients who have to linger in the hospital during the period between the two fractions. Thus, delivering external-beam PBI once per day might be a good option. The IMPORT LOW trial, in which PBI was delivered as 40 Gy in 15 daily fractions, demonstrated similar or fewer late adverse effects for PBI compared to WBI (30). As mentioned above, in the phase 3 Florence trial, the women in the PBI arm receiving 30 Gy in five daily fractions over 2 weeks with IMRT, had better QoL than those in the WBI arm (11). Other options for PBI, such as IORT or brachytherapy, require complicated and invasive techniques and are therefore not widely applied. Taking account of the low number of linear accelerator machines, high patient numbers, and the popularity of external-beam RT in China, we evaluated the once per day regimen with IMRT in this study. The present analysis showed that this regimen of PBI could provide excellent QoL and might be suitable for Chinese patients with early-stage breast cancer.

In our study, the analysis of changes in QoL over time showed that the scores for fatigue and pain significantly increased at the end of treatment compared to those at baseline in the PBI arm, which suggested that these two symptoms might be influenced more by PBI. The scores for dyspnea increased significantly after treatment and did not remarkably improve at the 1-year follow up, the reasons for which are unknown. The scores for future perspective at the end of RT were significantly lower than those at baseline in the PBI arm, indicating that PBI-treated patients worried about their disease. Breast symptom scores significantly increased at the end of RT and at the 6-month follow-up compared to the baseline scores in both the PBI and WBI arms, and arm symptom scores significantly increased at the 6-month follow-up in the PBI arm, which might reflect the side effects on the breast due to local RT. The analysis of QoL in the GEC-ESTRO trial (12) showed that the breast symptom scores markedly increased after treatment in both arms, followed by a decrease after 3 months, whereas arm symptom scores remained stable in both arms. Meattini and colleagues (11) reported that in the phase 3 Florence trial, breast and arm symptoms worsened in the WBI arm at the end of RT, and the scores remained significantly higher than the baseline scores at the 2-year follow-up. On the other hand, breast symptom scores did not change after PBI and improved significantly at the 2-year follow-up, and arm symptom scores remained stable throughout. At the 1-year follow-up in our study, most symptom and functional subscale scores that were influenced by RT recovered to levels similar to those before RT. Furthermore, the scores for the role and social functioning and financial difficulties subscales improved significantly compared to those at baseline, indicating that most treatment-related effects on QoL were transient and recovered over the course of 1 year, which was in line with the findings of other longitudinal QoL analysis of external-beam RT (31, 32). Similarly, in a prospective longitudinal analysis of 151 patients with PBI using high-dose-rate interstitial brachytherapy, Garsa et al. reported that scores for emotional functioning and financial difficulties significantly improved at 2 years after treatment (32).

There were several limitations of this study. Firstly, the enrolled patients were not masked to the allocated treatment, which might influence the patient-reported outcomes due to their expectations. Secondly, the statistical power might be impaired because QoL was not the primary endpoint of this randomized study and the sample size was small. Thirdly, the follow-up time is short, and the findings may not reflect long-term QoL outcomes. Finally, this study only enrolled Chinese patients, therefore the results may be different when applying to other patient populations, and the data of breast size was not collected which may play an important role in the breast-related QoL.

## Conclusion

Patients treated with PBI using the IMRT technique have comparable QoL outcomes with those treated with WBI. Compared with baseline scores, most QoL subscale scores that were influenced by RT would return to a similar or better level within 1 year after treatment, except for the dyspnea subscale scores. PBI with IMRT delivered in 10 daily fractions might be considered as a treatment option for selected cases of low-risk breast cancer after breast-conserving surgery.

## Data Availability Statement

The raw data supporting the conclusions of this article will be made available by the authors, without undue reservation.

## Ethics Statement

The studies involving human participants were reviewed and approved by National Cancer GCP for Anticancer Drugs, The Independent Ethics Committee, National Cancer Center/Cancer Hospital, Chinese Academy of Medical Sciences and Peking Union Medical College. The patients/participants provided their written informed consent to participate in this study.

## Author Contributions

Y-CS and G-YS: Formal analysis, investigation, data collection, methodology, and writing of the first draft. HF: Patient care and review and editing of the manuscript and revision of the manuscript. YuT: Statistical analysis review and editing of the manuscript. Y-WS: Patient care and review and editing of the manuscript. CH: Statistical analysis guidance. S-NQ: Patient care and review and editing of the manuscript. BC: Patient care and review and editing of the manuscript. HJ: Patient care and review and editing of the manuscript. YuaT: Patient care and review and editing of the manuscript. JJ: Patient care and review and editing of the manuscript. Y-PL: Patient care and review and editing of the manuscript. N-nL: Patient care and review and editing of the manuscript. Y-XL: Formal analysis and data collection, validation, statistical guidance, and project administration, patient care, and writing of the manuscript. S-LW: Formal analysis and data collection, validation, statistical analysis guidance, project administration, patient care, writing, and editing of the first draft of the manuscript. All authors contributed to the article and approved the submitted version.

## Funding

This study was supported by grants from the Capital Characteristic Clinic Project (Z171100001017116) and the National Natural Science Foundation of China (81972860).

## Conflict of Interest

The authors declare that the research was conducted in the absence of any commercial or financial relationships that could be construed as a potential conflict of interest.

## Publisher’s Note

All claims expressed in this article are solely those of the authors and do not necessarily represent those of their affiliated organizations, or those of the publisher, the editors and the reviewers. Any product that may be evaluated in this article, or claim that may be made by its manufacturer, is not guaranteed or endorsed by the publisher.
